# The effects of sarcolipin over-expression in mouse skeletal muscle on metabolic activity

**DOI:** 10.1016/j.abb.2015.01.027

**Published:** 2015-03-01

**Authors:** John Butler, Neil Smyth, Robert Broadbridge, Claire E. Council, Anthony G. Lee, Claire J. Stocker, David C. Hislop, Jonathan R.S. Arch, Michael A. Cawthorne, J. Malcolm East

**Affiliations:** a Centre for Biological Sciences, University of Southampton, Southampton SO17 1BJ, UK; b Peptide Protein Research Ltd, 184 Funtley Road, Fareham PO15 6DP, UK; c The Clore Laboratory, University of Buckingham, Buckingham MK18 1EG, UK

**Keywords:** Sarcolipin, SERCA, Thermogenesis, Energy expenditure, Obesity, High fat diet

## Abstract

Studies in sarcolipin knockout mice have led to the suggestion that skeletal muscle sarcolipin plays a role in thermogenesis. The mechanism proposed is uncoupling of the sarcoplasmic reticulum calcium pump. However, in other work sarcolipin was not detected in mouse skeletal tissue. We have therefore measured sarcolipin levels in mouse skeletal muscle using semi-quantitative western blotting and synthetic mouse sarcolipin. Sarcolipin levels were so low that it is unlikely that knocking out sarcolipin would have a measurable effect on thermogenesis by SERCA. In addition, overexpression of neither wild type nor FLAG-tagged variants of mouse sarcolipin in transgenic mice had any major significant effects on body mass, energy expenditure, even when mice were fed on a high fat diet.

## Highlights

• Sarcolipin is insufficient to affect thermogenic activity of SERCA in mouse muscle.
• The ratio of SLN to SERCA in total limb skeletal muscle is <0.0015 mol/mol.
• Knocking out this SLN in mice would have a small effect on SERCA function.
• Overexpressing SLN in transgenic mice only resulted in 0.037 mol SLN/mol SERCA.
• SLN^+/+^ mice showed no evidence of an increase in thermogenesis.

## Introduction

Sarcolipin is a 31aa transmembranous peptide found in the sarcoplasmic reticulum (SR)^1^ of skeletal muscle and cardiac atria that is believed to play a role in the modulation of SR calcium pumps (SERCAs) [1,2]. In addition to altering the kinetics of calcium transport, *in vitro* studies indicate that sarcolipin can partially uncouple calcium transport from ATP hydrolysis by these pumps, causing enhanced thermogenesis [3,4].

In a recent sarcolipin knockout (SLN^−/−^) study in mice, it was reported that sarcolipin was protective against hypothermia and obesity induced by a high fat diet; these effects were attributed to muscle-based thermogenesis, through the influence of sarcolipin on SERCA [5,6]. These findings are in line with the thermogenic effects of sarcolipin on the activity of SERCA observed *in vitro* [4]. However, an analysis of sarcolipin levels in the skeletal muscles of a range of mammals indicated that while large mammals such as pigs and rabbits have relatively large amounts of sarcolipin in their skeletal muscle, in smaller species, such as mice and rats, sarcolipin was undetectable [7]. Although sarcolipin has been detected in mouse skeletal muscle in other studies (see [5,8,9]), the levels of sarcolipin have not been quantified. This suggests that sarcolipin may have a role in muscle-based thermogenesis in larger mammals, whereas in smaller mammals its role in thermogenesis is less clear. It has also been reported that while sarcolipin ablation increased the stoichiometry of calcium uptake/ATP hydrolysed by SERCA from mouse soleus, supporting a role for sarcolipin in thermogenesis, the resting oxygen consumption by soleus was unaffected by sarcolipin ablation [10].

We have sought to resolve the discrepancy in the detection of sarcolipin in mouse muscle by using semi-quantitative western blotting to estimate the ratio of sarcolipin to SERCA in the SR of mouse skeletal muscle from mouse strains C57BL/6, the strain used in the sarcolipin knockout study [5], and FVBN, which is used here to produce transgenic mice over-expressing sarcolipin. In addition, in view of the previous study indicating that sarcolipin levels are undetectable in mouse skeletal muscle [7], which suggests that there is little value in knocking out sarcolipin, we have examined the effects of over-expressing sarcolipin and an epitope-tagged (FLAG) variant of sarcolipin in mouse skeletal muscle on high fat diet-induced obesity and energy expenditure.

## Methods

### Animals

All procedures were conducted in accordance with the University of Southampton and University of Buckingham Home Office UK project licences under the Animals (Scientific Procedures) Act (1986).

### Production of transgenic mice

A DNA sequence encoding mouse sarcolipin was obtained as described previously [11]. The mouse sequence was amplified by the polymerase chain reaction (PCR) using oligonucleotides atggaaaggtctactcaagagctg and agatcagtactggtaagaccgcactag to produce a sequence coding for the wild type protein, and aaggacgacgacgacaaggaaaggtctactcaagagctgttc and atggactacaaggacgacgacgacaaggaaaggtctac to produce a sequence coding for sarcolipin tagged with the FLAG epitope [12] at its N-terminus. The resulting PCR products were ligated into plasmid pSKMHSApA (provided by Dr. Edna Hardeman, University of New South Wales, Australia) consisting of the human skeletal actin (HSA) promoter, a splice acceptance site and two SV40 polyadenylation signal sequences [13]. Recombinants were cloned and sequenced and the insert flanked by HSA promoter and polyadenylation signals was released with NotI, gel purified and injected into FVB/N embryos. Both the sarcolipin founder and FLAG-SLN founder were bred to homozygosity to enhance the levels of transgene expression.

### Animal procedures

Mice were fed on a standard chow diet that contained 10% fat, 70% carbohydrate and 20% protein by energy (RM1; Beekay Feed, B&K Universal Ltd., Hull, UK), until they were 11 weeks old and then on a high fat diet that contained, by energy, 60% fat, 20% carbohydrate and 20% protein (D12492; Research Diets, New Brunswick, USA). They were housed at 22 °C with lights off from 07.00 to 19.00 h. Fourteen male mice of each genotype (wild type FVBN, *Sln^+^/^+^*, and FLAG-tagged *Sln^+^/^+^*) were housed in pairs. Food intake for each cage of mice was measured over 24 h when the mice were 10, 11 and (after being on the high fat diet for a week) 12 weeks old. Body weight was measured weekly. Energy expenditure was measured by open circuit indirect calorimetry in their home cages as described previously [14] at 10 weeks of age when the mice were eating standard chow and again at 26 weeks of age after they had been switched to the high fat diet.

### Preparation of sarcoplasmic reticulum from mouse skeletal muscle

SR was prepared using a modification of a method previously described [15]. The procedure was carried out at 4 °C. Skeletal muscle stripped from the fore and hind limbs was chopped and homogenised using a glass/Teflon homogeniser in 4 volumes of 0.1 M NaCl, 10 mM MOPS-Tris, pH 7.0. The homogenate was centrifuged at 8300*g* for 20 min and the supernatant centrifuged for a further 30 min at 12,000*g* for 30 min. The resulting supernatant was centrifuged at 53,000*g* for 40 min and the pellet was resuspended in 40 volumes of 0.6 M KCl, 5 mM Tris-maleate, pH 6.5 and stirred for 40 min. The suspension was then centrifuged at 125,000*g* for 45 min; the pellet was rinsed in 0.1 M sucrose, 30 mM MOPS-Tris, pH 7.0 then resuspended in the same buffer, snap frozen in liquid nitrogen and stored at −70 °C. Protein was measured using a DC protein assay kit (Bio-Rad).

### Semi-quantitative western blotting

Mouse skeletal muscle was homogenised in 300 mM sucrose, 10 mM imidazole pH 7.0 with the addition of protease inhibitor cocktail 1:100 (Sigma). Protein concentration was determined using a DC protein assay (Biorad). Homogenates were snap frozen and stored at −80 °C. All SDS–PAGE was carried out using 10–20% tricine gels (Invitrogen). The protein was transferred to PVDF membranes 0.2 micron pore size (GE Healthcare) and blocked overnight in 20 mM Tris, 150 mM NaCl (TBS) containing 5% bovine serum albumin (BSA). Western blotting of sarcolipin was carried out using either a primary antibody raised to sarcolipin peptide sequence VRSYGY in rabbit (a gift from Dr. Muthu Periasamy, Ohio State University) used at 1:1000 or from Millipore (Anti-Sarcolipin Antibody ABT13). Western blotting of SERCA was performed using the pan-SERCA rabbit polyclonal antibody (a gift from Peter Vangheluwe, K.U. Leuven, Belgium). Primary antibodies were detected using an IRDye goat polyclonal anti rabbit 800 nm conjugated antibody 1:10,000 (LI-COR) or IREDye goat polyclonal anti mouse 800 nm conjugate antibody 1:10,000 (LI-COR). The blots were detected and analysed using the LI-COR ODYSSEY detection system.

### Immunolocalistion of FLAG-SLN

Mouse skeletal muscle was snap frozen and sectioned (6 μm) using a Cryostat. Sections were collected onto poly l-lysine coated glass slides; fixed in acetone −20 °C for 10 min; washed in 137 mM NaCl, 2.7 mM KCl, 10 mM Na_2_HPO_4_, 2.0 mM KH_2_PO_4_, 0.1% Triton X-100, pH 7.4 (PBST) and blocked for 1 h in PBST containing 10% BSA. Anti-FLAG-FITC conjugate antibody was incubated at 1:100 (Abcam 1259) and rabbit polyclonal anti-SERCA 1/2/3 incubated at 1:100 (Santa Cruz Biotechnology, H-300 SC-30110) overnight at 4 °C. The primary antibodies were washed off with PBST and the anti SERCA primary antibody was detected using anti-rabbit Texas red conjugate raised in donkey 1:100 (GE Healthcare). The secondary antibody was washed off with PBST and the tissue incubated with DAPI stain (5 mg/ml diluted 1:5000 for working strength). Tissue was washed and then mounted using Mowiol mountant containing 0.1% citifluor anti-bleaching agent. Samples were viewed with a Leica TCS SP2 confocal microscope under oil with a 40× objective and pinhole diameter of Airy 1. Leica LCS software was used for image acquisition and analysis. Fluorescein was excited at a wavelength of 490 nm and emission was measured between 500 and 600 nm and Texas Red was excited at 594 nm and emission was measured between 605 and 700 nm.

### Statistics

Body weight, food intake and energy expenditure data were analysed by one-way analysis of variance at each age, followed by Dunnett’s multiple comparison test. Bartlett’s test showed no significant differences in variances between the three genotypes. A repeated measures two-way analysis of variance followed by Bonferroni post-tests with the control group was also conducted on the body weight data for animals from 10 to 28 weeks of age, and on the food intake measurements.

## Results and discussion

The study of Vangheluwe et al. [7] indicates that the levels of sarcolipin in mice are below the level of detection. Therefore, in this study, instead of knocking out the sarcolipin gene, transgenic mice were created using constructs under the control of the human skeletal actin promoter in order to over-express wild type mouse sarcolipin and FLAG-tagged sarcolipin in mouse skeletal muscle [13]. The FLAG tag was employed so that it would be possible to validate sarcolipin incorporation into to the sarcoplasmic reticulum; sarcolipin antibodies are ineffective in immunofluorescence microscopy.

In addition, using synthetic sarcolipin and semi-quantitative western blotting, the levels of sarcolipin expression were determined in the skeletal muscle of transgenic animals as well as in wild-type mice. The wild-type strains were both FVBN (used in this study) and C57BL/6 (used for the sarcolipin knockout studies reported previously [5,6]).

**Fig. 1:**
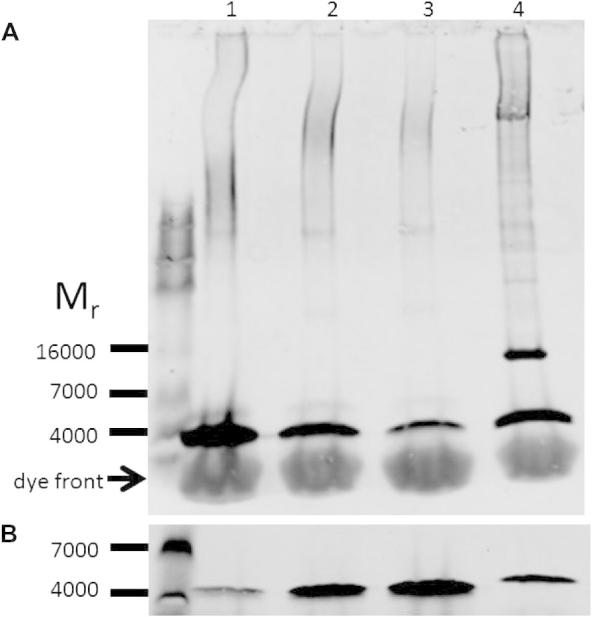
(A) Characterisation of sarcolipin levels in mouse atria and rabbit skeletal muscle by semi-quantitative western blotting. 30 μg (protein) of homogenised pooled left and right atrial tissue from 8 to 10 week old FVBN mice was separated by SDS–PAGE (lane 4) and synthetic sarcolipin, 20, 5.0 and 2.5 ng of peptide were included as standards (lanes 1, 2 and 3 respectively). Following the transfer of the proteins from the gel to PVDF membranes the blots were probed with anti-sarcolipin antibody, followed by a goat anti-rabbit fluorophore conjugated antibody. (B) SR, 1 μg, from a 3 kg New Zealand white rabbit was separated by SDS–PAGE (lane 4) and synthetic sarcolipin, 20, 40 and 60 ng (lanes 1–3). The blots were visualised and analysed using the LI-COR ODYSSEY detection system. Blots shown are typical of at least two determinations.

The semi-quantitative western blotting method using synthetic mouse sarcolipin was validated by estimating the levels of sarcolipin in the atria of FVBN mice. Fig. 1A is a western blot showing a comparison of the content of sarcolipin in 30 μg of mouse atria in lane 4 with 25, 5.0 and 2.5 ng of synthetic sarcolipin (lanes 1–3 respectively). Sarcolipin migrates by SDS–PAGE with an apparent *M*_r_ of 4000. The amount of sarcolipin in 30 μg of atrial tissue is approximately 9 ng. Using the estimate of SERCA obtained by western blotting of 14 μg/mg atrial tissue (data not shown), which is comparable to the value of 10 μg SERCA/mg atrial tissue obtained by [7]), this gives a molar ratio of 0.50 mol sarcolipin/mol SERCA. This value is similar to that previously reported: 1.24 mol sarcolipin/mol SERCA [7]. Similarly the blot shown in Fig. 1B compares 1 μg of rabbit total skeletal muscle SR (lane 4) with 20, 40 and 60 ng synthetic mouse sarcolipin (lanes 1–3 respectively). One microgram of SR contains approximately 27 ng sarcolipin. These data give a value of 1.2 mol sarcolipin/mol SERCA, assuming that rabbit muscle SR contains 70% SERCA (estimated from SDS–PAGE of SR stained with SYPRO orange). This is similar to the values previously reported for rabbit soleus and extensor digitorum longus muscles of 0.87 and 0.37 mol sarcolipin/mol SERCA respectively [7]. Even though the sequences of rabbit and mouse sarcolipin are different, the use of an antibody against the mouse sarcolipin sequence to quantify the rabbit homologue is justified because the antibody is raised against the peptide VRSYQY, corresponding to the C-terminal sequence of sarcolipin from numerous mammalian species, including mouse, rabbit and human.

**Fig. 2:**
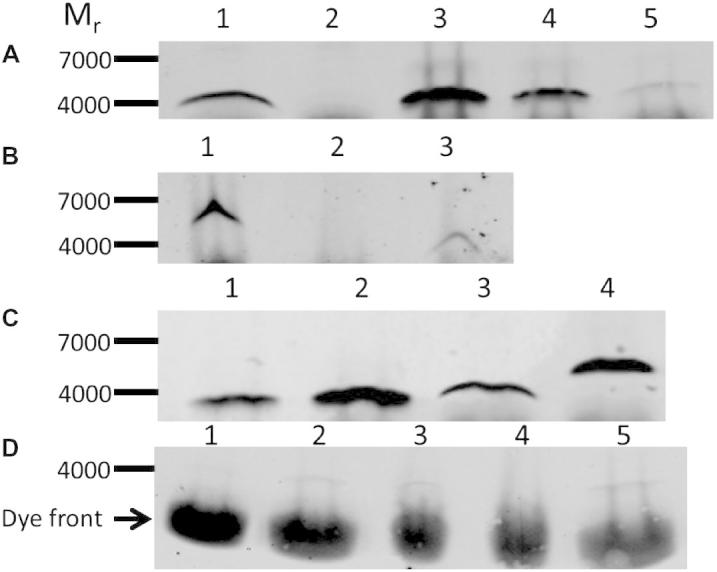
Expression of sarcolipin and FLAG-tagged sarcolipin in the skeletal muscle of transgenic and control mice detected by western blotting. (A) 40 μg (protein) of sarcoplasmic reticulum from sarcolipin^+/+^ transgenic mice and FVBN control mice (from total limb skeletal muscle) were separated by SDS–PAGE (lanes 1 and 2, respectively). Synthetic sarcolipin, 10, 5 and 1 ng of peptide were included as standards (lanes 3, 4 and 5, respectively). (B) 100 μg of homogenised total limb skeletal muscle from FLAG-tagged sarcolipin^+/+^, C57BL/6 and sarcolipin^+/+^ mice were separated by SDS–PAGE (lanes 1, 2 and 3, respectively). (C) 40 μg (protein) of sarcoplasmic reticulum from sarcolipin^+/+^ and FLAG-tagged sarcolipin^+/+^ transgenic mice (from total limb skeletal muscle) were separated by SDS–PAGE (lanes 3 and 4, respectively). Synthetic sarcolipin, 20 and 40 ng of peptide were included as standards (lanes 1 and 2). All mice used for the analyses above were aged 8–10 weeks (D) 120 μg (protein) of homogenised soleus muscle from 30 week old FVBN mice was separated by SDS–PAGE; mice were fed on the high fat diet from week 11–30 weeks (lane 3) or a standard chow diet throughout (lane 4). Synthetic sarcolipin, 1.0, 2.0 and 3 ng of peptide were included as standards (lanes 1, 2 and 5, respectively). In all cases, following the transfer of the proteins from the gel to PVDF membranes the blots were probed with anti-sarcolipin antibody, followed by a goat anti-rabbit fluorophore conjugated antibody. The blots were visualised and analysed using the LI-COR ODYSSEY detection system. Blots shown are typical of at least two determinations.

An examination of the sarcolipin levels in sarcoplasmic reticulum from total skeletal muscle of FVBN mice confirmed the finding of Vangheluwe et al. [7] that sarcolipin was below the level of detection in mouse skeletal muscle using western blotting techniques (Fig. 2A; lane 2) though as little as 1 ng of synthetic sarcolipin was detectable in this blot (lane 5). By contrast, the western blot procedure was able to detect sarcolipin in the SR of transgenic FVBN mice over-expressing this protein (lane 1). Sarcolipin knockout studies have been reported using C57BL6 mice [5,6,10], so the level of sarcolipin in this strain of mice was also examined. Sarcolipin was undetected by western blotting in homogenates of combined limb skeletal muscle taken from this mouse strain (Fig. 2B, lane 2), but FLAG-tagged sarcolipin and sarcolipin were readily detectable in muscle homogenates of transgenic FVBN mice over-expressing these proteins (lanes 1 and 3 respectively). The FLAG-tagged sarcolipin migrated under SDS–PAGE with an apparent *M*_r_ of around 6000. Previously, studies have shown that sarcolipin can be detected in soleus muscle from C57/BL6 mice [5,8] however the study by Bombardier et al. [10] clearly shows that the sarcolipin content of 100 μg of soleus is far less, by some considerable margin, than that contained by 1 μg of atrial tissue. Given that limb skeletal muscles such as white gastrocnemius, extensor digitorum longus and quadriceps contain little or no sarcolipin, presumably sarcolipin is diluted to such an extent that it is difficult to detect when C57/BL6 limb muscle is combined as in Fig. 2B. Soleus appears to be one of the limb skeletal muscles containing the highest levels of sarcolipin in mouse [8,9,16]. In this study the level of sarcolipin in 120 μg of homogenised soleus from 30 week old male FVBN mice was not detectable (Fig. 2D, lane 4) and was certainly less than 1 ng sarcolipin, which is just detectable (lane 1). Using the value for SERCA in soleus homogenates determined by western blotting of 21 μg SERCA/mg protein (data nor shown), which is comparable with the value from [7] of 16 μg SERCA/mg protein, the sarcolipin level in soleus is <0.012 mol sarcolipin/mol SERCA. Although Bombardier et al. [10] were able to demonstrate the presence of sarcolipin in wild type soleus this was close to the limits of detection, requiring an enhancement of the image. As previously described [6], the high fat diet did increase the level of sarcolipin in mouse soleus muscle. In Fig. 2D (lane 3) 120 μg of homogenised soleus from 30-week-old male FVBN mice fed on a high fat diet (lane 3) contained around 2 ng sarcolipin (2 ng sarcolipin standard in lane 2), which translates to 0.023 mol sarcolipin/mol SERCA.

These data indicate that the level of sarcolipin in mouse muscle is so low as to make it likely that any direct effect of sarcolipin on SERCA function [3,4] would be small and hence the effect of knocking it out would be difficult to detect.

The levels of mouse sarcolipin and FLAG-tagged sarcolipin in SR obtained from transgenic animals were calculated from the western blot shown in Fig. 2C. Using the sarcolipin standards in lanes 2 and 3 (20 and 40 ng sarcolipin respectively) and a value of 50% SERCA in mouse SR estimated from SDS–PAGE gels of SR stained with SYPRO orange (data not shown), the sarcolipin/SERCA ratios were estimated to be 0.037 mol sarcolipin/mol SERCA and 0.059 mol FLAG-tagged sarcolipin/mol SERCA. The levels of these transgenically-expressed proteins are still low compared to the levels in mouse atria and rabbit SR, where the value is close to 1 mol sarcolipin/mol SERCA.

**Fig. 3:**
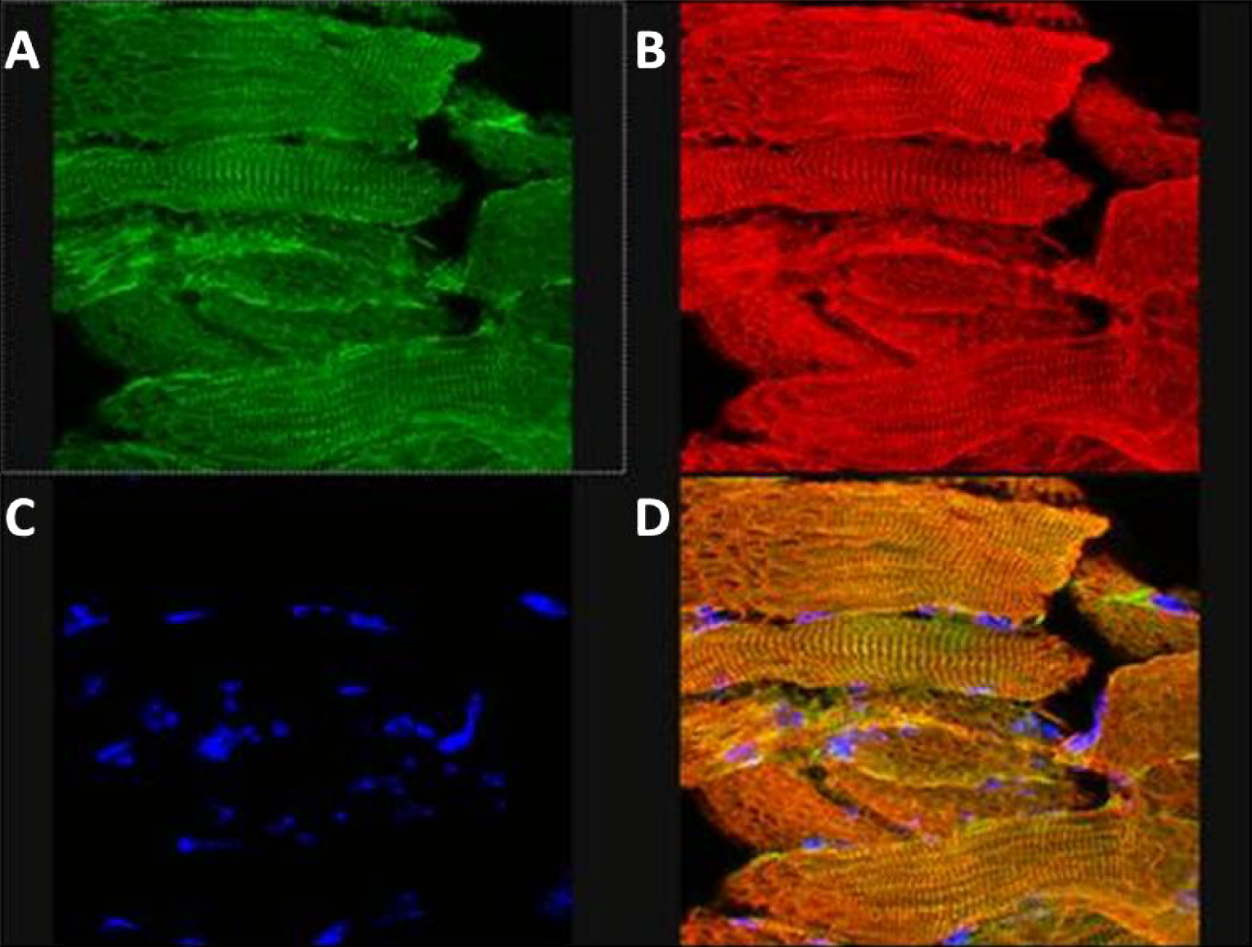
Immunolocalisation of SERCA and FLAG-tagged sarcolipin in mouse skeletal muscle. Frozen sections of skeletal muscle from transgenic mice expressing FLAG-tagged sarcolipin were acetone fixed, blocked for 1 h in PBST containing 10% BSA. The sections were then incubated with Anti-FLAG-FITC conjugate antibody (1:100) rabbit polyclonal anti-SERCA1/2/3 (1:100) (Santa Cruz). The primary antibodies were detected using donkey anti-rabbit Texas red conjugate (1:100). Following washing the section was incubated with DAPI stain 1 μg/ml and mounted in Mowiol containing 0.1% citifluor. FITC fluorescence is shown in panel A; Texas red fluorescence in panel B; DAPI fluorescence in panel C and FITC, DAPI and Texas red merged in panel D.

Fig. 3 shows that FLAG-tagged sarcolipin was co-localised with SERCA; the pattern of labelling was similar to that reported previously for the related SERCA modulatory peptide phospholamban [17], indicating that the FLAG-tagged sarcolipin was, and by inference the untagged construct would be, appropriately targeted to the SR.

**Fig. 4:**
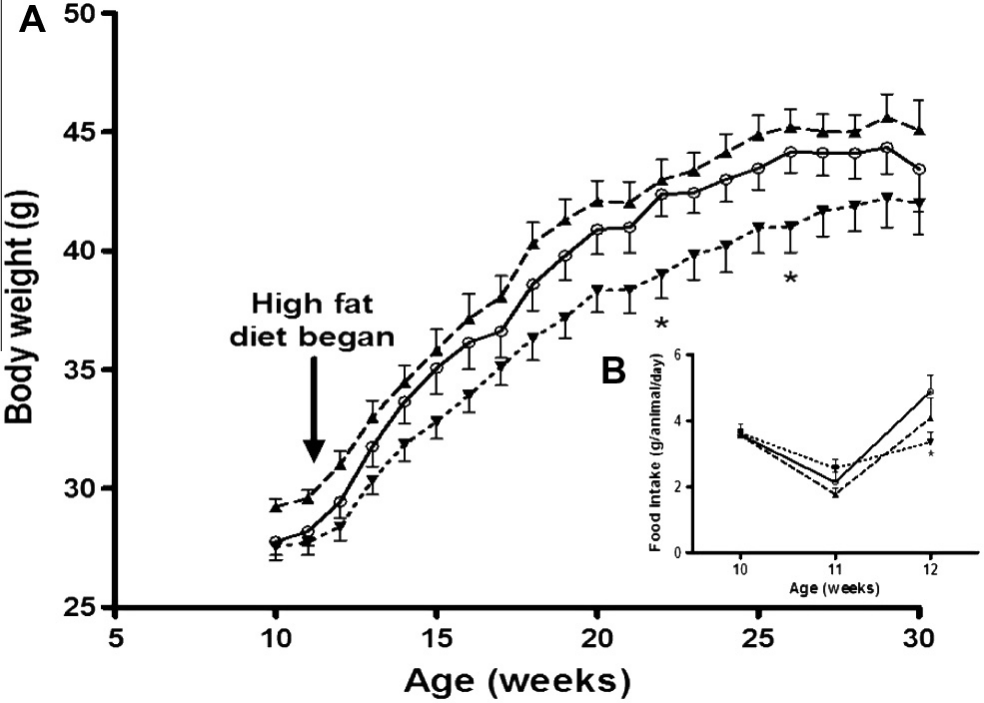
Effect of high fat diet on the body weight of sarcolipin over-expressing and FLAG-tagged sarcolipin expressing mice. (A) Male wild type (**○**, solid line), sarcolipin over-expressing (**▴**, dashed line) and FLAG-tagged sarcolipin expressing (**▾**, dotted line) mice were fed on a standard chow diet until 11 weeks of age after which they were transferred onto a high fat (60%) diet. Body weight was measured on a weekly basis. The bars indicate standard error of the mean, *n* = 14. ^∗^*P* < 0.05 for FLAG-sarcolipin overexpressing mice compared to control mice by one-way ANOVA followed by Dunnett’s test at 22 and 26 weeks of age. (B, inset) Food intake was measured during the transition from standard chow to high fat diet. The bars indicate standard error of the mean, *n* = 14. ^∗^*P* < 0.05 for FLAG-sarcolipin overexpressing mice compared to control mice by two-way ANOVA followed by Bonferroni multiple comparisons test.

Since sarcolipin has been shown to cause the uncoupling of the SERCA with which it is associated [3,4], one might predict that the over-expression of sarcolipin would result in animals with a higher energy expenditure, a reduced body mass and the potential to mitigate the effects of a high fat diet. However, Fig. 4 shows that there was no significant difference in body masses between the control animals and those expressing sarcolipin or FLAG-sarcolipin transgenes at 10 or 11 weeks after birth (fed on standard chow). After the animals were transferred to a high fat diet from 11 weeks of age, FLAG-sarcolipin expressing animals tended to gain less weight compared with control animals and SLN expressing mice, and at 22 and 26 weeks of age their body weight was significantly lower than that of the control group. However, this difference was not apparent at 30 weeks of age. Sarcolipin expressing mice mirrored the control animals when fed the high fat diet, showing no significant difference. Two-way analysis of variance of body weights showed an effect of genotype on body weight (*P* < 0.05) and an interaction between time and genotype (*P* < 0.01), consistent with this observation, but Bonferroni tests did not show significant differences between genotypes at any age. Two-way analysis of variance of body weight gain (data not shown) also showed a significant effect of genotype (*P* < 0.05), and in this case Bonferroni tests did show significant differences between the control and FLAG-SLN expressing animals, but only at 22 and 26 weeks of age (*P* < 0.05 at both ages). There was no significant difference between the food intake of the three groups before transfer onto the high fat diet, but repeated measures two-way ANOVA showed a significant interaction between time and genotype and a Bonferroni test showed that food intake was significantly lower (*P* < 0.05) in the FLAG-SLN expressing than the control animals after they were transferred to the high fat diet (Fig. 4 inset), consistent with reduced weight gain in these animals.

**Fig. 5:**
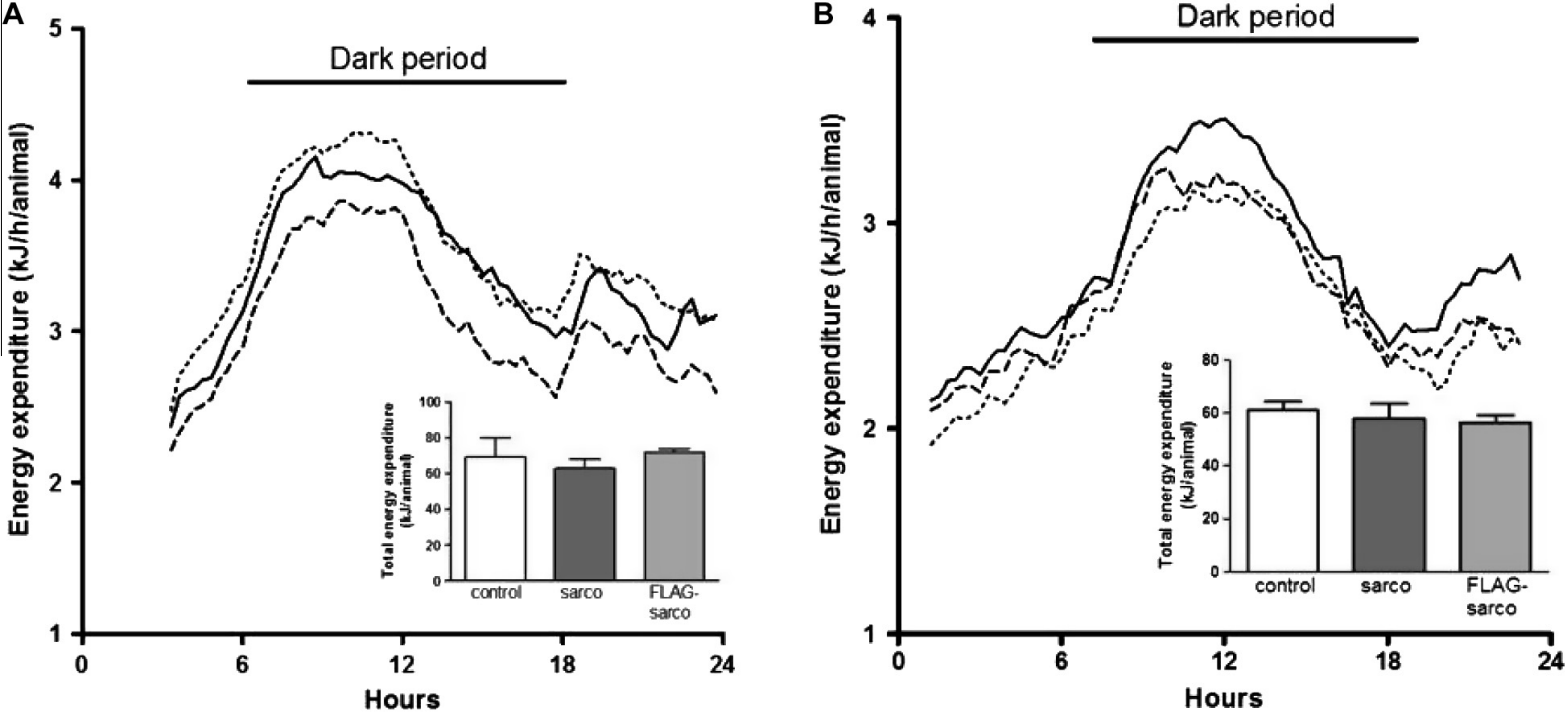
Effect of sarcolipin over-expression and FLAG-sarcolipin expression by skeletal muscle on metabolic rate. Hourly energy expenditure 1 week before (A) and 15 weeks after (B) the introduction of the high fat diet in male wild type (solid line), sarcolipin over-expressing (dashed line) and FLAG-tagged sarcolipin expressing (dotted line). Error bars have been omitted for clarity since the objective is to show that the diurnal rhythm of energy expenditure was similar in all genotypes Total energy expenditure over 24 h at 10 weeks of age and over 22 h at 26 weeks of age is shown in (A inset) and (B inset) respectively; *n* = 7 cages. There were no significant differences in energy expenditure at any hourly time point or in total.

The finding that there are no major effects of over-expressing sarcolipin and flag-tagged sarcolipin on body mass as a result of overfeeding is also reflected in the finding that energy expenditure, measured by open circuit indirect calorimetry, was similar for both wild type and transgenic animals (Fig. 5). There were no significant differences, either at 10 weeks of age (before the feeding of the high fat diet) (Fig. 5A) or after 15 weeks on the high fat diet (Fig. 5B), between wild type mice and mice over-expressing sarcolipin or FLAG-tagged sarcolipin in either the diurnal rhythm (main Fig. 5A and B) or in total energy expenditure (inset Fig. 5A and B), whether data were expressed for the whole animal (as in Fig. 5) or relative to body weight (data not shown).

Using the data provided by the *in vitro* investigation of the effects of sarcolipin on heat production [4] it is evident that, if all of the sarcolipin in the sarcolipin overexpressing mouse (0.037 mol sarcolipin/mol SERCA) were bound to SERCA, Δ*H* would rise from approximately 5.9 kcal/mol hydrolysed to 6.0 kcal/mol, i.e., a 1.7% increase in heat output. It is unlikely that such an effect would be detectable.

Using the same argument, it is also unlikely that effects reported for sarcolipin knockout mice, such as the loss of protection from both hypothermia and obesity, induced by a high fat diet [5] can be directly attributed to the direct effect of sarcolipin on the heat output of SERCA. The amount of sarcolipin in the skeletal muscles of these animals is so small (<0.0015 mol sarcolipin/mol SERCA for FVBN mice and virtually undetectable in homogenates of total skeletal muscle of C57BL6 mice) as to make the impact of its removal on the heat production by SERCA close to zero. The cause of the effect of sarcolipin knockout on thermogenesis in [5] is unclear, but may be related to more subtle effects on calcium signalling.

A definitive demonstration of the effects of sarcolipin on energy expenditure *in vitro*, acting through a direct effect on, will require a knockout experiment employing an animal, such as rabbit, which has significant levels of skeletal muscle sarcolipin. Knocking out sarcolipin in rabbit skeletal muscle (from this study the value is 1.2 mol sarcolipin/mol SERCA) would result in a reduction in heat output from approximately 7.6 kcal/mol to 5.9 kcal/mol (a drop in heat output of 22% calculated using the data in [4]). Alternatively, the creation of a transgenic mouse expressing near stoichiometric amounts of sarcolipin relative to SERCA in skeletal muscle would provide a useful comparator.

## Footnotes

1 *Abbreviations used*: PBST, phosphate buffered saline plus Triton X100; SDS–PAGE, sodium dodecyl sulphate polyacrylamide gel electrophoresis; SR, sarcoplasmic reticulum; DAPI, 4′,6-diamidino-2-phenylindole; HRP, horseradish peroxidase.
